# Hydrogen production by a fully *de novo* enzyme†

**DOI:** 10.1039/d4dt00936c

**Published:** 2024-06-13

**Authors:** Sigrid Berglund, Clara Bassy, Ibrahim Kaya, Per E. Andrén, Vitalii Shtender, Mauricio Lasagna, Cecilia Tommos, Ann Magnuson, Starla D. Glover

**Affiliations:** a Physical Chemistry, Department of Chemistry, Ångström Laboratory, Uppsala University Box 523 SE-75120 Uppsala Sweden Starla.glover@kemi.uu.se; b Department of Pharmaceutical Biosciences, Spatial Mass Spectrometry, Science for Life Laboratory, Uppsala University Box 591 SE-75124 Uppsala Sweden; c Division of Applied Materials Science, Department of Materials Science and Engineering, Uppsala University 75103 Uppsala Sweden; d Molecular Biomimetics, Department of Chemistry, Ångström Laboratory, Uppsala University Box 523 SE-75120 Uppsala Sweden; e Department of Biochemistry and Biophysics, Texas A&M University College Station TX 77843 USA

## Abstract

Molecular catalysts based on abundant elements that function in neutral water represent an essential component of sustainable hydrogen production. Artificial hydrogenases based on protein-inorganic hybrids have emerged as an intriguing class of catalysts for this purpose. We have prepared a novel artificial hydrogenase based on cobaloxime bound to a *de novo* three alpha-helical protein, α_3_C, *via* a pyridyl-based unnatural amino acid. The functionalized *de novo* protein was characterised by UV-visible, CD, and EPR spectroscopy, as well as MALDI spectrometry, which confirmed the presence and ligation of cobaloxime to the protein. The new *de novo* enzyme produced hydrogen under electrochemical, photochemical and reductive chemical conditions in neutral water solution. A change in hydrogen evolution capability of the *de novo* enzyme compared with native cobaloxime was observed, with turnover numbers around 80% of that of cobaloxime, and hydrogen evolution rates of 40% of that of cobaloxime. We discuss these findings in the context of existing literature, how our study contributes important information about the functionality of cobaloximes as hydrogen evolving catalysts in protein environments, and the feasibility of using *de novo* proteins for development into artificial metalloenzymes. Small *de novo* proteins as enzyme scaffolds have the potential to function as upscalable bioinspired catalysts thanks to their efficient atom economy, and the findings presented here show that these types of novel enzymes are a possible product.

## Abstract 2

Artificial hydrogenases based on designed protein-inorganic hybrids are a promising venue to improve catalysis for sustainable fuel production.

## Graphical abstract

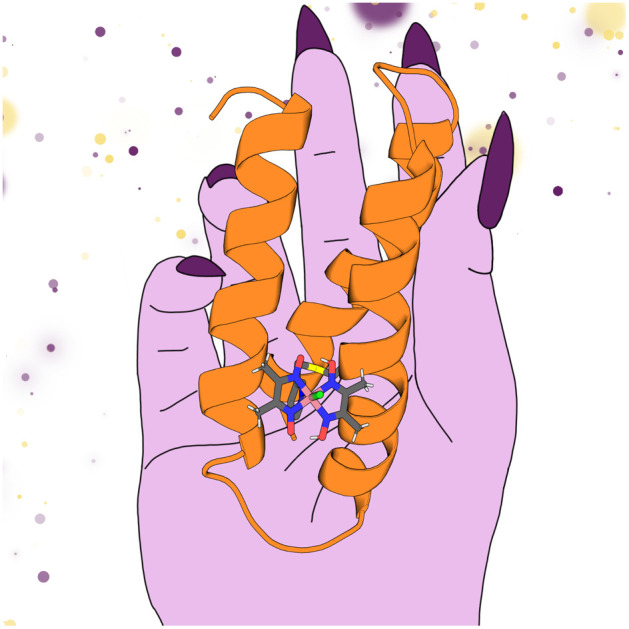

## Introduction

In this time of global warming and resulting climate crises, discovery of sustainable energy carriers is an important venture not only for researchers in science and technology but for all of humanity. One path to the sustainable production of fuels is through the use of molecular, metal-based catalysts for the hydrogen evolution reaction (HER) and CO_2_ reduction; advances in this field of research are ongoing.^1,2^ Several major challenges must be overcome before the use of molecular catalysts to produce fuels is feasible on a large scale. The foremost challenge is to design molecular catalysts that are composed of earth-abundant elements and can operate at mild overpotentials with respect to the target chemistry. Additionally, molecular catalysts are frequently only soluble, functional and stable in organic solvents, which is not sustainable in the long term. Production of organic solvents contributes to global warming, due to their derivation from fossil precursors, and by evaporation to become greenhouse gases.^3^

To overcome the above challenges, current efforts focus on hydrogenase enzymes, and other molecular catalysts that are water-soluble, based on abundant elements, and that function at mild overpotentials. Hydrogenases are proteins that catalyse the reversible reduction of protons to molecular hydrogen. Several classes of hydrogenases exist, the unifying theme being that they have a catalytic site containing at least one transition metal ion, most notably iron. A variety of other metals may be employed in synthetic molecular HER catalysts. Cobaloxime, [Co(dmg)_2_Cl_2_], where dmg = dimethylglyoxime, is a cobalt-based HER catalyst that first received attention after it was described as a vitamin B12 mimic.^4,5^ The dmg ligands bind cobalt in a square planar fashion, which structurally mimics the corrin macrocycle of cobalamin. More than four decades ago, the possibility of cobaloxime as an HER catalyst was explored by the chemical reduction of aqueous Co(dmgBF_2_)_2_ using Cr^2+^, Eu^2+^ and V^2+^.^6^ The low stability of cobaloxime in particularly aqueous solution has challenged investigations, and presently a majority of studies involving cobaloximes as HER catalysts have been carried out in organic solvents, or solvent-water mixtures, where cobaloxime activity decreases with increasing water concentrations.^7,8^ Recent efforts have been made to expand the use of cobaloxime to aqueous systems primarily through engineering of the secondary coordination sphere, either by designing complex axial and planar ligands or by incorporating the cobaloxime in a secondary framework, such as proteins and other organic matrices.^9–14^

In this work, we studied catalytic hydrogen evolution in a new, cobaloxime-functionalized *de novo* protein. The protein scaffold is based on a single chain of 65 amino acids that folds into a structurally well-defined three alpha-helical (α_3_) bundle motif.^15^ Cobaloxime was coordinated to the α_3_ protein scaffold *via* a cysteine residue at position 32, resulting in a cobalt-containing artificial hydrogenase with HER activity that we demonstrated by chemical, electrochemical, and photochemical reduction. Our strategy aims to overcome two of the three challenges for molecular catalysts, specifically to increase operability in water solution and the use of abundant materials. The α_3_C protein (C = cysteine) is part of the family of α_3_X proteins, where X denotes the amino acid at site 32.^16–22^ The family of α_3_X proteins have been designed to contain both natural and unnatural amino acids at site 32, such as tyrosine, tryptophan, mercaptophenol, or fluorotyrosine.^16–22^ In α_3_C, a cysteine resides at position 32, which lies at a buried position on the interior of the protein. Previous studies with the α_3_X homologs have shown several advantageous properties that make them an excellent choice for redesign into artificial enzymes. The α_3_X proteins display only minor changes in their secondary and tertiary structures over a broad pH range (∼5.5–10), which facilities mechanistic and catalytic studies. Solution NMR structures are available for four members of the α_3_X protein family.^16–18,23^

The α_3_ scaffold is electrochemically and photochemically nonlabile except for the dedicated redox site at position 32.^21^ Previous studies have shown that tyrosine and tryptophan radicals generated at site 32 in α_3_Y and α_3_W persisted on surprisingly long timescales.^18,20^ The long lifetime for reactive radical species can be attributed to (i) the redox inactivity of the α_3_ scaffold (*i.e. E*′°(X_32_) ≪ *E*′° of all other residues) and (ii) the well-defined protein structure that blocks rapid radical decay by radical–radical dimerization. Additionally, the UV/Vis absorption properties of reduced and radical states of several α_3_X proteins are known.^18,20,23^ The redox-inert and well-defined structural and spectroscopic properties of α_3_X makes this protein system a promising choice for further development into an artificial hydrogenase.

Mass spectrometry, cyclic voltammetry, and electron paramagnetic resonance (EPR) and circular dichroism (CD) spectroscopies were used to demonstrate the successful incorporation of cobaloxime in the protein scaffold, and the maintained protein folding in the presence of ligated cobaloxime. Our HER assays show a minor decrease in the amount of hydrogen produced in the cobaloxime-functionalized *de novo* protein, compared to molecular cobaloxime in the aqueous phase.^10,12^ The results of this study underscore the challenge and complexity of designing artificial enzymes even when the precursors are well-characterised.

## Results and discussion

### α_3_X redesign into an artificial hydrogenase

**Fig. 1:**
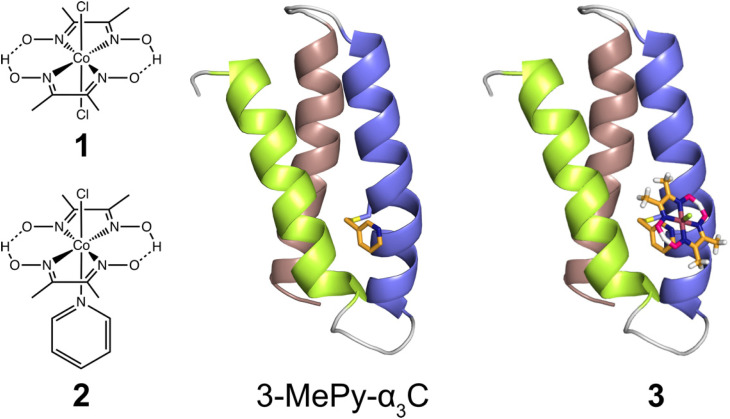
Left: Cobaloximes used in the present study: [Co(dmgH)_2_Cl_2_] (1), and [Co(dmgH)_2_(py)Cl] (2). Center: A structural model of 3-MePy-α_3_C (3-MePy = 3-methyl pyridine) based on the related protein system 2MP-α_3_C (2MP = 2-mercaptophenol), RSCB PDB ID 2LXY.^17^ Sidechains are omitted for clarity. Helix 1, 2, and 3 of the α_3_ scaffold is shown in lime green, blue, and taupe, respectively. Right: A model of the artificial hydrogenase, 3, based on 3-MePy-α_3_C.

In the present study, we use the cysteine residue of α_3_C to link cobaloxime to the α_3_ scaffold to impart hydrogenase activity. Starting from purified α_3_C, the cobaloxime functionalized protein, 3, is prepared in two steps. First, the cysteine of α_3_C is labelled with 3-methylpyridine (3-MePy), to give 3-MePy-α_3_C, Fig. 1. In the second step, 1 and 3-MePy-α_3_C are stirred in an excess of TEA reducing agent; this leads to the loss of a chloride and coordination of 3-MePy to 1 to give 3, Fig. 1. In both steps α_3_ is denatured to expose the interior residues to the bulk, and the protein is subsequently refolded during purification by dialysis. Further details are provided in the Experimental section and ESI.†

### Characterisation of functionalized α_3_C

**Fig. 2:**
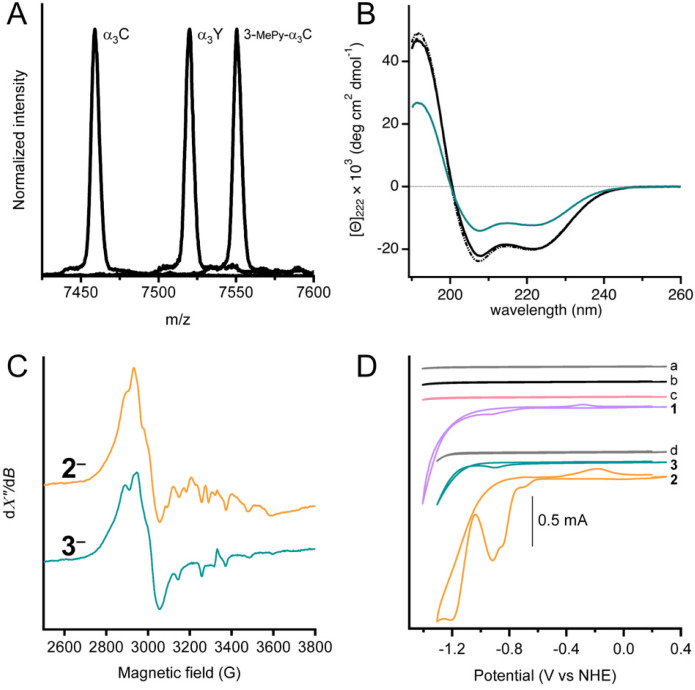
Characterization of complex 3. (A) MALDI-ToF-MS spectrographs of α_3_C, 3-MePy-α_3_C and α_3_Y using a sinapinic acid matrix. Calculated (*m*_c_) and observed (*m*/*z*) masses for each sample are as follows: α_3_C (*m*_c_ = 7460.8, *m*/*z* = 7458.9), 3-MePy-α_3_C (*m*_c_ = 7551.9, *m*/*z* = 7550.5), α_3_Y (*m*_c_ = 7520.8, *m*/*z* = 7520.1). (B) Circular dichroism spectra given in units of mean residue molar ellipticity, [Θ] for: α_3_W (black dot-dash), α_3_C (solid black), and 3 (teal) in 50 mM KP_i_, pH 7.0 ± 0.1. (C) X-band EPR spectra of frozen solutions chemically reduced samples, 2^−^ and 3^−^. Spectra recorded at *T* = 7 K and using 1 mW microwave power. (D) Stacked cyclic voltammograms of 100 mM KP_i_ pH 7 buffer (scan a, grey), protein film of α_3_C (scan b, black), protein film of 3-MePy-α_3_C (scan c, pink), 0.47 mM 1 (scan 1, purple), 100 mM MES, 100 mM MOPS, 75 mM KCl buffer (scan d, grey), protein film of 3 (scan 3, teal) and 1.0 mM 2 (scan 2, orange). CVs a–c and 1 were recorded in 100 mM KP_i_ pH 7.0 ± 0.1 buffer. CVs d, 2, and 3 were collected in 100 mM MES, 100 mM MOPS pH 7.0 buffer. A scan rate of 250 mV s^−1^ was used for all measurements.

MALDI-ToF-MS analysis was used to confirm covalent linking of 3-MePy to C32 in α_3_C (Fig. 2A). The measured *m*/*z* agrees well with the calculated masses (*m*_c_) for α_3_C (*m*/*z* = 7458.9 Da and *m*_c_ = 7460.8 Da) and 3-MPy-α_3_C (*m*/*z* = 7550.5 Da and *m*_c_ = 7551.9 Da). α_3_Y was used as a control with a measured *m*/*z* consistent with its theoretical mass (*m*/*z* = 7520.1 Da and *m*_c_ = 7520.8 Da). The MALDI-ToF-MS-spectrum of 3 showed a broadened peak, with several sharp features (Fig. S1†) between the calculated masses of 3 and 3-MePy-α_3_C. This is likely due to fragmentation of the cobalt complex upon laser desorption. The splitting features have been reported previously for other metal binding peptides, as well as synthetic metal coordination complexes.^24,25^ Quantification of independently prepared samples of 3 showed cobalt : protein ratios that ranged from 1 : 1.20 to 1 : 2.68 with an average ratio of 1 : 1.79. Because of the variation in ratios, the cobalt concentration was determined independently for every sample assayed by ICP-OES or colorimetric assay using 4-(2-pyridylazo)resorcinol.^26^ Details of these assays and a table of cobalt concentrations are provided in the ESI.†

UV-Visible spectra of α_3_C, 3-MePy-α_3_C, and 3, also informed on the functionalization at site 32 (Fig. S2†). The α_3_C spectrum showed an intense peak at 220 nm from the protein backbone and no absorbing features in the 240–290 nm range, which is consistent with the absence of aromatic side chains. The UV-vis spectrum of 3-MePy-α_3_C showed a peak at 260 nm consistent with the addition of pyridine to the protein. 3 shows a weak absorption maximum at around 240 nm, which is masked by the stronger absorption of pyridine.

Circular dichroism (CD) spectroscopy was used to quantify the α-helical content and global stability of the α_3_ scaffold when cobaloxime was bound to site 32. The solution NMR ensemble structure of α_3_W contains 51 ± 1 (78%) α-helical residues.^16,19^ The remaining 14 ± 1 residues are located at the N and C termini and in the loop regions between the α-helices are random coil, as expected. α_3_W, α_3_C and 3 exhibit strong spectral features consistent with an α-helical structure, specifically negative signals at 222 and 208 nm and a positive signal at 192 nm (Fig. 2B). The CD spectra of α_3_C and 3 are compared to α_3_W in units of mean residue molar ellipticity, [Θ]. When spectra are plotted using [Θ], the amplitude at 222 nm ([Θ]_222_) is proportional to the α-helical content.^19^ Comparison of [Θ]_222_ for α_3_W *versus* α_3_C shows that here is no significant difference in the α-helical content of these proteins. The CD spectrum of 3 shows a notable reduction in [Θ] relative to the α_3_W and α_3_C spectra (Fig. 2B). The drop in the [Θ]_222_ of 3 corresponds to a lowering in the α-helical content to about half of the 65 residues residing in an α-helical backbone configuration and the remaining half displaying random coil configurations. Perturbation of the α_3_C structure upon cobaloxime ligation may take the form of fraying, *i.e.*, breaking of the backbone N–H/C=O hydrogen bonds, at the ends of the three α-helices.

Chemical denaturation experiments were carried out to determine the global stability of α_3_C and 3 at pH 7 (Fig. S3†). Again, we use α_3_W as a well-characterized control. All three proteins exhibited very similar global stabilities of −4.8 ± 0.7, −4.3 ± 0.6, and −4.3 ± 0.4 kcal mol^−1^ for α_3_W and α_3_C, and 3, respectively. These numbers are close to earlier obtained values for α_3_W and other α_3_X proteins.^19,20,27^ The chemical denaturation curves are very similar for α_3_W and α_3_C, and as the concentration of urea approaches zero and 10 M the slope of the curve approaches zero. The shapes of the α_3_W and α_3_C denaturation plots are consistent with a monomeric protein going through a highly cooperative unfolding/folding transition. The shape of the denaturation curve for 3 is different with steeper slopes for the folded (at low [urea]) and unfolded (at high [urea]) states. The less cooperative unfolding/folding transition observed for 3*vs.* α_3_W and α_3_C is consistent with α-helical end fraying and an overall lower α-helical content.

X-band EPR spectroscopy was used to confirm the cobaloxime coordination and oxidation state. For the purpose of producing the paramagnetic Co(ii), samples of 2 and 3 were treated with [Eu(EGTA)]^2−^ as a reductant. The absence of an EPR signal in unreduced samples indicated that both complexes were initially prepared in the Co(iii) form. After reduction, both samples displayed an EPR spectrum (Fig. 2C) typical of low-spin Co(ii) with rhombic *g*-tensor and hyperfine anisotropy, similar to previously reported observations.^28–31^ It is not possible to resolve the individual *g*-values in the spectra in Fig. 2 as we have used X-band EPR spectroscopy only. However, previous studies have shown that the degree of *g*-tensor anisotropy highly depends on the axial ligand. The difference in anisotropic shift between *g*_*x*_ and *g*_*y*_ is small for cobaloxime with two axially coordinated pyridines, and the spectrum appears similar to that of an axial system (*g*_*x*_ = *g*_*y*_ = *g*_⊥_). Cobaloxime with a single coordinated pyridine has a larger separation between *g*_*x*_ and *g*_*y*_, with some overlap, while the spectrum displays considerable rhombicity for cobaloxime with no strongly coordinating axial ligands.^31^ In the spectra from 2 and 3, the *g*_*x*_ and *g*_*y*_ resonances overlap around 2900 G (*g* ≈ 2.3) but with significant line broadening which is likely due to *g*-tensor anisotropy, consistent with axial coordination of one pyridine per molecule. Notably, the two spectra are not identical and display differences in shape. The features owing to *g*_*x*_ and *g*_*y*_ are slightly more separated in complex 3 with a shift towards lower *g*-value on the high-field side around 3000 G. This difference indicates a slight difference in the ligand field of the Co(ii) ion in 3 compared to 2, and can be explained by a weaker, or less well defined, binding of the sixth Co(ii) ligand. The protein environment in 3 is expected to restrict access of solvent to the catalytic site more than for 2, and the effect on axial coordination of solvent molecules can therefore explain the change in *g*-value anisotropy.^31^ The *g*_*z*_-value is close to that of the free electron in both 2 and 3, but it is difficult to determine due to the Co hyperfine structure. In conclusion, these spectra support our assertion that the Co(ii) center is in a protein environment in 3.

Fig. 2D shows cyclic voltammograms of buffer (scans a and d), apo proteins α_3_C (scan b) and 3-MePy-α_3_C (scan c), [Co(dmgH)_2_Cl_2_] (scan 1), [Co(dmgH)_2_(py)Cl] (scan 2), and cobaloxime functionalized protein 3, (scan 3). The voltammograms of the buffers and apoproteins do not show any significant faradaic current, indicating the absence of redox activity within the potential window investigated. Cyclic voltammetry of freely diffusing 1 and 2 displayed similar features to what has been previously reported for cobaloximes in water.^13,32^ The voltammogram of 2 showed an irreversible peak at *ca.* −0.92 V and a larger irreversible signal with an onset at −1.05 V *vs.* NHE; both signals have been attributed to HER activity.^13^ The protein film voltammogram of 3 showed features similar to that of 2: an irreversible peak at −0.90 V and a larger signal with an onset of −1.05 V *vs.* NHE. Scans for 2 and 3 were normalized to the irreversible peak at ca −0.9 *vs*. NHE (Fig. S4†), which shows that the irreversible peak and onset potential for the steep rise in current beyond the first reduction are nearly coincident for 2 and 3. The small anodic peak shift of 20 mV in 3 may be a reflection of the difference in electron donating properties when the pyridine *meta* position has a –CH_2_S *versus* an –H group; as –CH_2_S is not expected to be strongly deactivating in the *meta* position very similar peak potentials between 3 and 2 are expected. Notably, the similarity in features between 2 and 3 confirmed that the electrochemical behavior of cobaloxime is maintained when coordinated to pyridine in the α_3_ scaffold.

### Photocatalytic hydrogen production

**Fig. 3:**
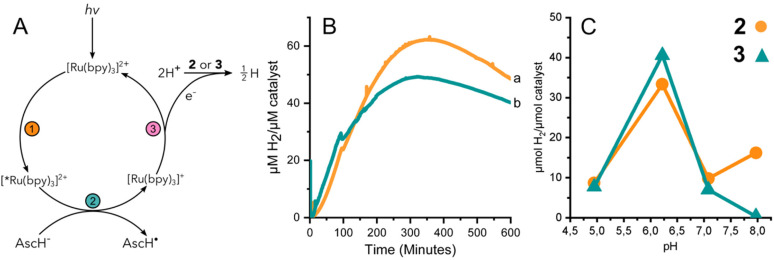
(A) Reaction scheme for photoactivated HER. (B) H_2_ produced by 2 (a, orange) and 3 (b, teal) at pH 6.2 under photocatalytic conditions. Detection by H_2_ sensing electrode. (C) H_2_ detected in headspace at reaction completion under a range of pH conditions.

The performance of 3 to yield hydrogen by means of photocatalysis was investigated by illuminating solutions containing 2 or 3, the photosensitizer [Ru(ii)(bpy)_3_]^2+^, and a sacrificial electron donor, ascorbic acid. Fig. 3A summarizes the light activated reductive quenching process that produces the reductant, [Ru(i)(bpy)_3_]^+^, by illumination of the reaction mixture. Based on the electrochemical characterization of 2 and 3, [Ru(i)(bpy)_3_]^+^ is sufficiently reducing, to drive catalysis.^33^ Two equivalents of reductant are utilized by either 2 or 3 to reduce protons from the solvent to hydrogen.

Fig. 3B shows the continuous detection of dissolved hydrogen by a Clark-type electrode submerged in illuminated reaction mixtures containing 2 or 3 at pH 6.2; the maximal amount of hydrogen detected was 62 and 49 μM H_2_ per μM catalyst, respectively. In other words, the hydrogen evolved by 3 was approximately 80% of that of complex 2. Independent experiments were further carried out for 2 and 3 at different pH values. The amount of accumulated hydrogen was quantified after about 15 hours using gas chromatography (GC). The hydrogen that was collected in the headspace for 2 and 3 at different pH values shows a clear maximum at pH 6 (Fig. 3C). The data suggest that 3 produces a slightly higher amount of hydrogen than 2 at pH 6, specifically 41 and 33 μM H_2_ per μM catalyst, respectively.

The effect of pH on the amount of hydrogen produced by photocatalysis is likely dominated by the behavior of the catalyst and not the [Ru(bpy)_3_]^2+^/ascorbate photosensitizer/quencher system. The luminescence quenching efficiency and electron recombination rates for [Ru(i)(bpy)_3_]^+^/AscH˙ in aqueous media have been reported for a pH range of *ca.* 3–8.^34^ Above the p*K*_a_ of ascorbic acid (p*K*_a_ = 4.5) the rate of luminescence quenching and fraction of quenched excited states were found to be nearly constant, and the rate constant of recombination between [Ru(i)(bpy)_3_]^+^ and the ascorbate radical anion was smaller by a factor of two at pH 6–7 compared to at pH 4–5. The analysis suggested that a steady state concentration of [Ru(i)(bpy)_3_]^+^ available to deliver reducing equivalents to the catalyst is expected to be relatively stable at pH values >4.5. In the present study we have taken great care to use equal concentrations of buffer, [Ru(ii)(bpy)_3_]^2+^, and ascorbic acid and illumination for the different photocatalytic assays. Thus, the greater production of hydrogen by both 2 and 3 at pH 6 is likely due to the properties of cobaloxime in the presence of water. The significantly lower activity of 2 and 3 at pH 5 is consistent with instability that has been reported previously for cobaloximes.^13,35^ At higher pH, protonation of cobaloxime to form the intermediate hydride becomes less favourable, which would result in reduced hydrogen production.^36,37^ Therefore, intermediate pH values are the most favourable, yielding the highest activity.

### Hydrogen evolution using a chemical reductant

**Fig. 4:**
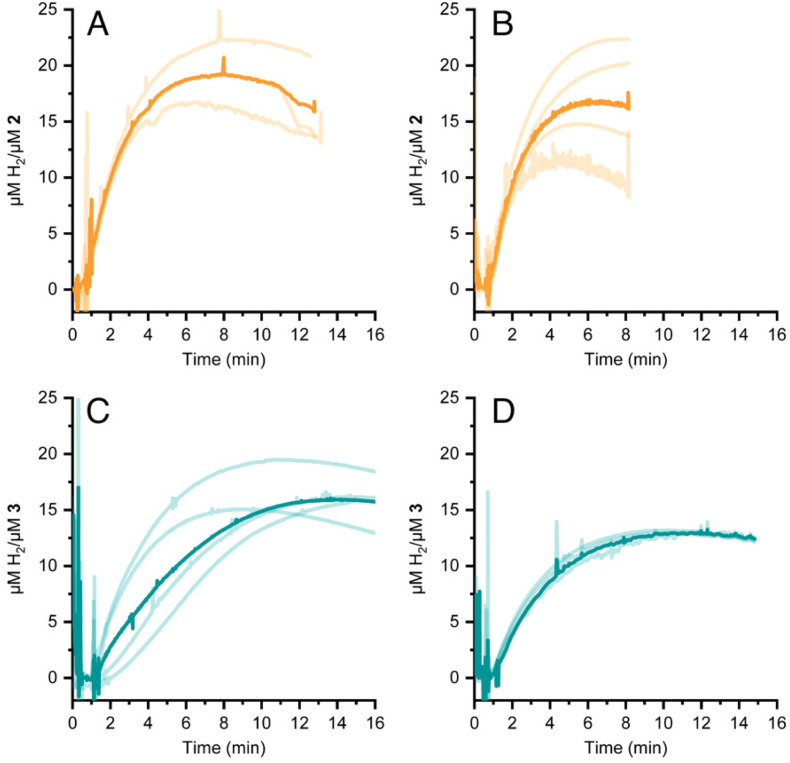
HER by 2 (panels A and B) and 3 (panels C and D) induced by addition of the chemical reductant [Eu(EGTA)]^2−^ The pH was either 7 (panels A and C) or 8 (panels B and D) Each light trace represents an independent assay; the bold trace is the average of light traces.

HER activity by 2 and 3 was measured at pH 7 and 8 in the presence of the strong chemical reducing agent [Eu(EGTA)]^2−^, *E*°[Eu(EGTA)]^2−/−1^ = −0.88 V *vs*. SHE at pH 8.^38^ An investigation at pH 6 was not possible due to poor solubility of EGTA at this pH. The amount of dissolved hydrogen was detected using a Clark-type hydrogen microsensor. Fig. 4A–D show the hydrogen evolved in independently prepared samples of 2 (panels A and B) or 3 (panels C and D) that were injected with a >19 fold excess of [Eu(EGTA)]^2−^. Each light trace corresponds to a single assay of an independently prepared sample; the bold trace in each panel is the average of the independent measurements. Table 1 summarizes the turnover numbers (TON) and rates associated with hydrogen production that were observed for 2 and 3 at pH 7 and 8.

**Table 1:** Turnover numbers (TON)^a^ and time passed in minutes (min), and hydrogen evolution rates^d^ in μmol H_2_ per μmol catalyst per min for 2 and 3 detected by a hydrogen sensor immersed in the reaction mixture

pH	TON (min) 2	H_2_ evolution rate 2	TON (min) 3	H_2_ evolution rate 3
6 b	62 (354)	0.32	49 (320)	0.35
7 c	18 ± 3 (8)	6.0 ± 0.5	16 ± 2 (13)	2.5 ± 0.5
8 c	16 ± 5 (5)	7.8 ± 0.9	13 ± 0.2 (10)	3.2 ± 0.3

a The turnover number (TON) was taken from plateau of curves of μmol H_2_ per μmol catalyst from Fig. 4 and 5.

b From photochemical hydrogen production.

c Average and standard deviation from 3–4 independent measurements.

d Rates were determined from slopes of HER curves. See Fig. S5 and S6† for details.

Under chemically reducing conditions the HER rates were 1.3 fold faster at pH 8 than at pH 7. The result was unexpected given that the higher proton concentration at pH 7 should facilitate HER. This effect can be explained by the equilibrium behavior between free Eu(ii) and [Eu(EGTA)]^2−^ at different pHs. The [Eu(EGTA)]^2−^ complex forms when the ligand is fully deprotonated, EGTA^4−^. EGTA ligand has four protonatable groups; at lower pH the EGTA ligand becomes increasingly protonated, which leads to a larger fraction of uncoordinated Eu(ii). Using methods described in ref. 34 and a stability constant of 9.38 for [Eu(EGTA)]^2−^, it is possible to calculate the concentration of [Eu(EGTA)]^2−^ as a function of pH.^39–41^ Solutions from each assay investigated contained 0.1 mM EuCl_2_ and 0.1 mM EGTA; taking into account pH equilibria of the complex, the concentration of [Eu(EGTA)]^2−^ is 1.3 fold greater at pH 8 *versus* pH 7 (0.097 *versus* 0.075 mM, respectively). The observed HER rates for 2 and 3 were 1.3 fold faster at pH 8 *versus* pH 7. This numerical agreement suggests the observed HER rate has a first order dependence on [Eu(EGTA)]^2−^, though additional data is needed to show a such a dependence definitively. If the observed HER rates are first order in [Eu(EGTA)]^2−^, then under the present experimental conditions HER is pH independent, which may again be due to kinetic limitations, *e.g.* diffusion of reductive equivalents to the catalyst.

Comparisons in catalytic performance of 2 and 3 are described in the general discussion, below.

### General discussion

The aim of this study was to develop a fully artificial enzyme for hydrogen production and quantify its catalytic behaviour. Cobaloxime and α_3_C were chosen for the development of this artificial enzyme on the basis that both have structural and redox-active properties that are well understood. With respect to the structure of 3, the CD analyses indicate that binding cobaloxime to the pyridine of 3-MePy-α_3_C changes the alpha helical folding with respect to α_3_C. To identify specific changes that occur, more extensive structural characterization, *e.g.* by 2D NMR spectroscopy, is needed. However, based on solution NMR ensemble structures of related α_3_C proteins functionalized with 2- and 4-mercaptophenol (2MP and 4MP, respectively),^17,23^ it is possible to predict how the cobaloxime may be situated in the α_3_X scaffold. The NMR structure of 2MP-α_3_C shows that the phenol ring is buried in the interface of helices 1 and 2. Structures of 2MP-α_3_C and 4MP-α_3_C show that the mercaptophenol functional group is directed to the exterior of the protein, yet has an average solvent accessible surface areas (SASA) of ∼4% and ∼9%, respectively.^23^ The small SASA is consistent with the mercaptophenol being a buried residue. Given the structural similarity between the mercaptophenol and methylpyridine, there is a high likelihood that the pyridyl ring is also buried between helices 1 and 2 of α_3_C. Upon binding to the pyridine of 3-MePy-α_3_C, cobaloxime is expected to be situated in a partially buried position between helices 1 and 2. Fig. 5 shows the protein model of 3 with protein surface including α_3_ amino acid side chains for 3 and shows that cobaloxime is likely nested between helices 1 and 2 in a partially solvated environment.

**Fig. 5:**
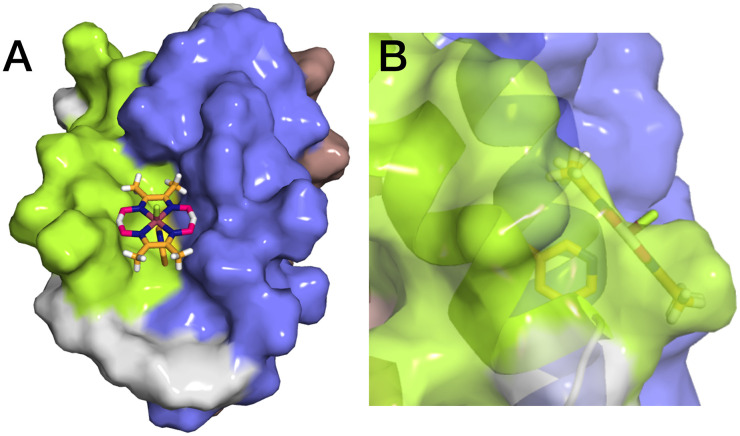
Surface representations of the α_3_ scaffold calculated in PyMOL for the α_3_ scaffold in 3, *cf*. Fig. 1. (A) An overhead view of site 32 showing the cobaloxime at site 32 sits between helix 1 (lime green) and helix 2 (blue). (B) Side-on view of site 32 through helix 2 showing that cobaloxime is partially buried in the protein scaffold.

Based on the quantitative data for 2 and 3 under the conditions investigated, the cobaloxime-functionalized *de novo* protein, 3, produces somewhat less hydrogen and at slower rate than free cobaloxime, 2. Using the TON from the photocatalytic and chemical reduction experiments as a metric, it was found that 2 produced *ca.* 10–20% more hydrogen than 3 under the conditions studied. The exception is at pH 6 where the hydrogen detected by GC from the endpoint titration was slightly greater for 3 than 2 (Fig. 3C). Rates of hydrogen production under chemically reducing conditions with [Eu(EGTA)]^2−^ were 2.4-fold faster in 2*versus*3 at both pH 7 and 8, while the initial rates of hydrogen production under photochemical conditions were significantly slower, and nearly identical for 2 and 3.

The lower HER rate under photochemical reduction, compared to chemical reduction, is likely due to lower steady-state availability of the reductant, which limits the bimolecular electron transfer between [Ru(bpy)_3_]^+^ and the catalyst. This conclusion is supported by the similarity in the photocatalytic HER rates between 2 and 3 under our experimental conditions. The protein scaffold in 3 can be expected to slow down the intermolecular electron transfer from the photosensitizer to the catalyst compared to cobaloxime in solution. However, the low availability of photogenerated [Ru(bpy)_3_]^+^ limits catalytic turnover, masking differences in electron transfer rate. By using a chemical reductant, the excess of reducing equivalents precludes this limitation and permits resolution of different turnover rates in hydrogen production between 2 and 3.

The slower HER rate for 3 can therefore be explained by the different solvation environments. Free cobaloxime, 2, is fully exposed to the bulk solution whereas the cobaloxime of 3 is flanked by glutamate and lysine residues that introduce steric bulk in the outer coordination sphere of the active site. In PCET studies of tyrosine in α_3_Y, MD simulations showed that local protein fluctuations reorient amino acid sidechains close to site 32; these fluctuations open a pathway for transient access of water into the hydrophobic protein core.^22^ The α_3_ protein scaffold of 3, will also be subject to local protein fluctuations which can permit or restrict transient access of substrate protonated water. It can also be that neighbouring amino acids in 3 interfere with the proton relay behavior of the equatorial glyoxime ligands. In contrast, 2 lacks protein and is not subject to any transient restrictions about the active site, nor possible interference from amino acid sidechains; free solvation in water without transient interference from protein can explain why 2 to produces hydrogen at a faster rate than 3.

Apart from structural and electrostatic differences of the catalyst environment, the cobalt centre may have slightly different electronic properties in 2 and 3 due to the difference in axial pyridyl ligands. The –CH_2_S group is *meta* with respect to the coordinating N on the pyridine of 3 is expected to be mildly deactivating, but the electronic contribution is expected to be very small. This was reflected in small shift to more positive onset potential for catalysis in 3 and would be expected to reduce catalysis to some degree, owing to the formation of a less basic Co–H species.^32^ The small electronic influence suggests that sterics have a much larger effect on the catalytic activity.

**Fig. 6:**
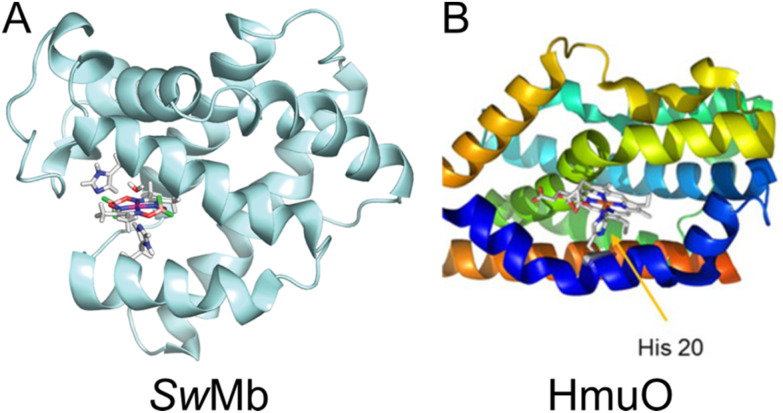
(A) Computed structure of cobaloxime in the binding pocket of sperm whale myoglobin (SwMb). Adapted with permission from ref. 12: Copywrite 2014 American Chemical Society. (B) Structure of heme oxygenase (HmuO) from *Corynebacterium diphteriae*. Adapted with permission from ref. 10: Copywrite 2016 Wiley-VCH Verlag GmbH &Co.

Cobaloxime has previously been incorporated into several natural protein systems.^9,10,12,42^ Studies of HER catalysis have been carried out for cobaloxime-modified versions of sperm whale myoglobin (SwMb),^10^ and in heme oxygenase from rat (HO) and *Corynebacterium diphteriae* (HmuO).^12^ SwMb, HO and HmuO are proteins with a native heme binding pocket, in which the heme group is axially coordinated to a histidine. In SwMb and HmuO, two cobaloximes, [Co(dmg)_2_(H_2_O)_2_] and [Co(dmgBF_2_)_2_(H_2_O)_2_], were introduced into the heme binding pocket (Fig. 6). Diagnostic CD spectra of cobaloxime labeled SwMb and HmuO were reported, and confirmed the presence of a-helical content, however, quantitative CD analyses to rule out significant perturbation from the native structure was not undertaken. In both studies the HER activity of [Co(dmgBF_2_)_2_(H_2_O)_2_] was significantly reduced or inactivated upon introduction to the protein environment. For the [Co(dmg)_2_(H_2_O)_2_] functionalized proteins, the SwMb variant was reported to have moderately increased HER activity (from 2.5 TON to 3.2 TON).^10^ For the heme oxygenase variants, however, the activity was approximately doubled (from 2.5 TON to 6.2 TON for HO and to 5 TON for HmuO); theoretical calculations of this system suggested that higher activity was attributed to a more flexible binding pocket in the heme oxygenases compared to myoglobin.^12^

The scaffold for 3, 3-MePy-α_3_C, is a *de novo* protein that does not contain a dedicated binding pocket nor does it contain histidine. The cobaloxime is instead coordinated to an unnatural, *in vitro* modified amino acid, 3-methylpyridine-cysteine. As we have suggested in this study, the cobaloxime in 3 is likely more exposed to the bulk in comparison to the SwMb- and HmuO-based complexes. The α_3_C scaffold is less likely to bury cobaloxime in its core, and the greater degree of solvent exposure to the catalyst in 3 compared to in SwMb, HO and HmuO could have a less hampering effect on the catalytic activity. On the other hand, the environment supplied by the protein surrounding cobaloxime in 3 is less well-defined, which may have a negative effect on the stability of the catalyst. SwMb, HO and HmuO, being proteins with native metal binding pockets, will readily accommodate non-native metal complexes in a stable and predetermined environment, which could make the catalyst less vulnerable to deleterious reactions.

The choice of axial ligand can have a significant impact on the catalytic activity and turnover frequency of cobaloximes.^13,32^ In a previous study, cobaloxime coordinated to imidazole, which is the side chain of histidine, was shown to yield a lower catalytic current than for cobaloxime coordinated to a thiol-pyridine.^32^ On the other hand, 4-methylpyridine, which is similar to 3-methylpyridine used for complex 3, was found to result in a relatively high catalytic current when compared to other axial ligands including basic pyridine, the axial ligand of complex 2.^32^ It might therefore be expected that the difference in axial ligand would lead to an increase in HER activity by complex 3 compared to complex 2, as well as the cobaloxime-protein conjugates based on SwMb and HmuO. This was not observed, however, indicating that other effects play a larger role for the activity than the chemical and electronic nature of the first coordination sphere.

## Conclusions

The α_3_C *de novo* protein scaffold has been successfully functionalized with the HER catalyst cobaloxime to investigate the effect of this molecular framework on catalytic activity. Complex 3 was characterised using CD, UV-vis and EPR spectroscopies, electrochemistry, and mass spectrometry. The protein-catalyst complex was a functional HER catalyst both by photocatalysis, using the photosensitizer [Ru(bpy)_3_]^2+^ and sacrificial quencher ascorbic acid, and by chemical reduction using the reductant [Eu(EGTA)]^2−^. Based on studies conducted here, the α_3_ scaffold appears to impair HER activity to a small degree. Under the photochemical and chemical reducing conditions it appears that intermolecular electron transfer from the reductant to the cobaloxime limits the rate of hydrogen production. In comparison, significantly faster rates of hydrogen production were demonstrated for cobaloxime adsorbed to photosystem I (PSI).^9^ It was determined that 2–4 non-covalently bound cobaloximes produced hydrogen at a rate of 350 mol H_2_ per (mol PSI) per min and a TON of 5200 after 100 minutes when illuminated in the presence of ascorbic acid. Even though cobaloxime was not covalently bound, the removal of diffusion and rapid intramolecular electron transfer of PSI gave rise to impressive rates of hydrogen production. In light of the studies with PSI, more efficient electron transfer to cobaloxime in 3, SwMb, and HmuO would likely increase rates of hydrogen production. In addition to optimized electron transfer, strategic placement of protonatable amino acids near the active site can assist proton transfer to improve catalytic rates; this much was observed in a series of cobaloximes that utilized an axial pyridine functionalized with tyrosine or phenylalanine.^13^ The protein-cobaloxime hybrids that have been investigated thus far indicate that there is a fine balance between a favourable electrostatic environment, and steric functionality of the protein structure that protects the integrity of the cobaloxime, which determines the ideal compromise between solvent accessibility and structural stability. Nature has evolved such a balance in hydrogenases, hydrogen producing enzymes, that enables reversible interconversion between hydrogen and protons at impressively high rates and efficiencies.^43^ Yet, natural hydrogenases may not be appropriate for future use in scaled-up systems due to their need for strict anaerobic conditions and poor atom economy. Small artificial enzymes for hydrogen production are, thus, a promising avenue for further development.^10,12,44^ Our work in this direction highlights the complexity of constructing artificial enzymes by rational design, and motivates the need for a deeper understanding of the synergy between protein frameworks and catalyst centres.

## Experimental section

### Chemicals

Chemicals were purchased from Merck-Sigma Aldrich, Strem Chemicals ([Ru(bpy)_3_]Cl_2_), and Nacalai Tesque, Inc., and used without further treatment. Modified pet32b-α_3_C and pet32b-α_3_W plasmids were obtained from Genscript and Novagen, respectively. *E. coli* strains Rosetta 2 BL21 (Novagen) and BL21(DE3) (Invitrogen) were used for recombinant protein expression to obtain α_3_C and α_3_W, respectively.

### Cobaloxime synthesis

Synthesis of [Co(dmg)_2_Cl_2_] (1) and [Co(dmg)_2_(py)Cl] (2), (Fig. 1) were carried out as described previously.^45,46^

### Expression and purification of α_3_C

Production of the α_3_C protein (C = cysteine at site 32) was performed as described previously by Tommos and co-workers,^17^ with the following differences. The expression system used was the BL21 derived Rosetta(DE3) *E. coli* strain, and protein expression was induced overnight in LB medium. DNAse was added to the harvested cells, and lysozyme treatment was omitted. The cells were lysed either by three freeze–thaw cycles in N_2(l)_ and 30 °C water, or by sonication.

### Covalent modification of cysteine in α_3_C

The α_3_C protein concentration was determined using the Pierce BCA protein assay (Thermo Scientific). The α_3_C protein was denatured with 2 M guanidinium hydrochloride in 25 mM potassium phosphate at pH 8–8.5. A 10-fold molar excess of 3-bromomethyl-pyridine was added to the protein solution. The reaction was gently shaken at room temperature for at least 5 hours but up to 20 hours. The resulting solution was pale yellow due to the formation of Br_2_ as the bromide is substituted by the cysteine sulphur (Fig. S7†). The product was dialysed against water using 3.5 kDa dialysis tubing to give purified 3-methyl-pyridine-α_3_C (3-MePy-α_3_C). Lyophilization of 3-MePy-α_3_C was performed for long term storage.

### Cobaloxime coordination to pyridine in 3-MePy-α_3_C to give 3

The method to coordinate [Co(dmg)_2_Cl_2_] to the pyridine functional group in 3-MePy-α_3_C was adapted from a previously described procedure.^11^ A solution of 3-MePy-α_3_C in 2 M guanidinium hydrochloride and 50 mM phosphate buffer, pH 6, was prepared. A 10-fold molar excess cobaloxime was pre-dissolved in methanol, DMSO or water, and then added to the 3-MePy-α_3_C solution. The 3-MePy-α_3_C : [Co(dmg)_2_]Cl_2_ solution was sparged with argon for 20 minutes to remove residual oxygen. At least a 45-fold molar excess of triethylamine (TEA) was added to the 3-MePy-α_3_C : [Co(dmg)_2_]Cl_2_ solution and the reaction incubated under inert atmosphere for at least 2 hours at room temperature with gentle stirring. The products were transferred to 3.5 kDa dialysis tubing and dialysed against water. The resulting solution of cobaloxime-3-methyl-pyridine-α_3_C (3) had the characteristic pale-yellow colour of dissolved cobaloxime. The dialysed protein was stored as a freeze-dried powder or in buffer at −20 °C.

### Optical spectroscopy

UV-vis absorption spectra were collected with a Chirascan VX (Applied Photophysics), Cary50 or Cary5000 spectrometer (Varian) using 1 cm, 4 mm, 2 mm or 1 mm quartz cuvettes (Starna). Circular dichroism spectra were collected on a Chirascan VX (Applied Photophysics) equipped with a temperature-controlled cell Peltier holder. Spectra were recorded in triplicate from 260–190 nm with a 1 s acquisition time per data point using a 2 mm cuvette and cell temperature of 25 °C. No smoothing was necessary owing to the low signal-to-noise observed for all recorded spectra. The spectra were converted from millidegrees to units of mean residue molar ellipticity [Θ] in deg cm^2^ dmol^−1^ by setting [Θ] = θ_obs_10^6^/*Cln*, where θ_obs_ is the recorded ellipticity in millidegrees, *C* is the concentration of protein in μM, *l* is the cuvette pathlength in mm, and *n* is the number of amino acids (*n* = 65 for all proteins investigated).

Protein global stability measurements involved chemical denaturation and were carried out as follows. A protein stock solution was prepared by dissolving lyophilized protein powder (α_3_W, α_3_C) or titrating small volumes of concentrated solution (3) into 50 mM KP_i_ pH 7 buffer to reach a raw ellipticity at 222 nm (θ_222_) of ∼−190 mdeg in a 1 mm quartz cuvette. The protein stock solution was then added to 50 mM KP_i_ pH 7 buffer containing 0 M or 10 M high purity urea (Nacalai Tesque, Inc.) to a 16.2-fold dilution of protein to give a θ_222_ of *ca.* −50 mdeg. The final concentration of urea was 0 and 9.5 M and the pH 7.0 ± 0.05 did not change upon the addition of protein, therefore no further pH adjustments were made. The concentration of urea was controlled by manual titration of different volumes of protein solutions containing 0 and 9.5 M urea to a final volume of 400 μL. Each sample was equilibrated for 3 minutes prior to spectral acquisition. Protein concentrations were determined by the Pierce BCA assay for the stock and diluted protein solutions with 0 M urea and standard curves for the Pierce BCA assay.

The method to determine the absolute α-helical content has been reported previously.^19,27^

### EPR spectroscopy

Samples containing 200 μL of 200 μM 2 and 116 μM 3 in 90 mM MOPS and 10% glycerol at pH 7 were chemically reduced using [Eu(EGTA)]^2−^. All manipulations were performed in an N_2_ atmosphere. [Eu(EGTA)]^2−^ was prepared as follows. Solutions of 3 mM EuCl_2_ and 3 mM EGTA in 100 mM MOPS pH 7 were prepared separately. [Eu(EGTA)]^2−^ was prepared by mixing equal volumes of EuCl_2_ and EGTA solutions to give 1.5 mM [Eu(EGTA)]^2−^. 1.1 molar equivalents of [Eu(EGTA)]^2−^ was titrated into the solution containing 2 or 3. A faint change in colour from pale-yellow to brighter yellow was noted upon addition of chemical reductant. The reduced samples were pipetted into EPR tubes and immediately frozen in an isopropyl alcohol bath externally cooled by liquid N_2_. Control samples of unreduced 2, 3 and 3-MePy-α_3_C were prepared under identical conditions. These samples showed no EPR signal.

The samples were stored in liquid N_2_ until the EPR measurements. CW EPR measurements were carried out with an X-band EPR spectrometer (Bruker E500-ELEXYS, Bruker GmbH, Germany) equipped with a rectangular 4102 standard cavity. Cryogenic temperatures were maintained using a continuous-flow cryostat and an ITC 503 temperature controller (Oxford Instruments). All spectra were recorded at 7 K using a 10 G modulation amplitude, 100 kHz modulation frequency, and a microwave power of 1 mW.

### Quantification of hydrogen production driven by chemical reduction

The hydrogen evolution reaction (HER) performed by 2 and 3 (using sample C described in Table S1†) was quantified using a Clark type H_2_ microsensor (Unisense, H_2_ needle sensor for piercing low range (NPLR)). The microsensor was calibrated by measuring the signal in air-saturated water by addition of increasing concentrations of dissolved H_2_.

The HER samples were prepared in a glove box under a N_2_ atmosphere. First 2 or 3 were dissolved in water and diluted into HEPES buffer to a final concentration of 100 mM HEPES, and a final concentration of *ca.* 3 μM catalyst. 4 mL of the solution containing 2 or 3 was transferred to a 9 mL vial, along with a small stir bar, and isolated from the atmosphere using a septum closure. Upon removal from the glove box, the H_2_ sensor needle was inserted through the septum and submerged in the catalyst solution.

The HER was provided reducing equivalents by chemical reductant [Eu^II^(EGTA)], which was prepared in an N_2_ glove box. Solution A contained 40 mM EuCl_2_ in 100 mM HEPES at pH 7 or 8 and solution B contained 80 mM EGTA 100 mM HEPES at pH 7 or 8. Immediately prior to the hydrogen evolution assay, the reducing [Eu(EGTA)]^2−^ solution was prepared by titrating 100 μL of solution A into 50 μL of solution B to give 26.7 mM [Eu(EGTA)]^2−^.

A baseline reading was collected for *ca.* 1 minute and then 15 μL of the freshly prepared [Eu^II^(EGTA)] solution was injected with an Ar-flushed Hamilton syringe. The measurement was terminated after a plateau was reached. H_2_ evolution experiments were repeated 3–4 times for 2 and 3 at pH 7 and 8. One additional set of measurements was made for 3 at pH 7; these additional data are given in the ESI.†

A fresh sample of [Eu^II^(EGTA)] was prepared for each assay. Precise concentrations of cobalt were quantified using ICP-OES after each experiment and are reported in the ESI, Table S2.†

### H_2_ quantification of photocatalytic hydrogen production

All samples were irradiated with a LED PAR38 lamp (17 W, 50 mV cm^−2^) having an intensity of 1 kW m^−2^ between 420 and 750 nm. The distance between the sample and the lamp was adjusted such that the power meter (ThorLabs PM100D power meter with a photodiode sensor) read 30–35 mW at 450 nm. Solutions for photocatalytic hydrogen production contained 150 μM [Ru(bpy)_3_]Cl_2_, and 100 μM ascorbate in 50 mM phosphate buffer, and 2 or 3, with concentrations between 2 μM and 15 μM. Hydrogen production was measured either by GC or by a calibrated Clark type H_2_ microsensor.

For measurements where H_2_ produced was measured by GC, 2 ml of photocatalytic solution was added to a 9 ml microwave vial, and the pH was adjusted using concentrated KOH or H_3_PO_4_. The vials were sealed with a septum and degassed by gentle argon sparging for 15 min under atmospheric pressure at room temperature. Samples were illuminated for 15 hours. 100 μL of headspace was removed with a gas tight syringe and quantified by GC, (Claris 500, PerkinElmer LLC). The hydrogen concentration was determined from comparison with a calibration curve.

When the H_2_ produced was measured by a microsensor: 2.3 mL of the photocatalytic solution was contained in a 10 × 4 mm quartz cuvette; the headspace was minimized. The cuvette was sealed with a septum and the sample was gently degassed with Ar. During irradiation samples were shielded by a 420 nm longpass filter to slow the degradation of [Ru(bpy)_3_]Cl_2_ by UV light.

### MALDI-TOF

Samples were prepared by mixing 3 μL protein solution sample with 3 μL matrix solution (20 mg ml^−1^ sinapinic acid in 50% acetonitrile/water, containing 0.2% trifluoroacetic acid) and vortexed briefly. The samples were spotted with 1 μL each on the MTP 384 ground steel target plate left for drying at room temperature. All the mass spectrometry data (mass range of 4–20 kDa) were acquired with an UltrafleXtreme MALDI TOF/TOF MS Instrument (Bruker Daltonics GmbH) running in linear positive (LP) mode at 1 kHz laser repetition rate, and with the laser beam focus set to ultra. External calibration of the method was performed using Bruker protein calibration standard I (mass range of 4–20 kDa). An autoXecute method in Flex control (v 3.0 Bruker, Daltonics GmbH, Germany) were used to collect 50 satisfactory laser shots in 40 shot steps from each spot on the MALDI MTP 384 ground steel BC target plate with random raster movement. The laser power was optimized at the start of each analysis and then held constant during the MALDI-ToF-MS experiment. Flex Analysis (v 3.0 Bruker, Daltonics GmbH, Germany) were used for MS data analysis. Average spectra were exported as ASCII file from Flex Analysis.

### Cyclic voltammetry

All cyclic voltammetry was performed using a potentiostat (Metrohm Autolab PGSTAT302N) connected to a PC running NOVA 2 using a three-electrode configuration. The working electrode was pyrolytic graphite edge (PGE), 3 mm (BASi). The counter electrode was a platinum rod (Metrohm). The reference electrode was Ag/AgCl (Metrohm) placed inside a salt bridge containing the sample buffer. All voltammetry measured a range of 0.1–1.6 V *vs.* Ag/AgCl (α_3_C, 3-MePy-α_3_C and 1) or 0.1–1.5 V *vs*. Ag/AgCl (2 and 3) under 90% *iR*-compensation using 250 mV s^−1^ scan. The voltammograms of 1 and of α_3_C and 3-MePy- α_3_C were collected in 100 mM potassium phosphate pH 7. The voltammograms of 2 and 3 were collected in 100 mM MES, 100 mM MOPS, 75 mM KCl.

For all samples involving protein, cyclic voltammetry was carried out on protein films. The working electrode surface was prepared by polishing with p1200 grit sandpaper until a completely matte surface formed and cleaned by prolonged rinsing with water then acetone. Protein films were prepared by drop casting 3 μL of water containing 200 μM α_3_C or 104 μM 3-MePy-α_3_C, or by dipping the electrode into 75 μM 3 (100 mM MES, 100 mM MOPS, pH 7) for 30 seconds where the sample concentration of 3 is reported with respect to the cobalt concentration. For α_3_C and 3-MePy-α_3_C, the water was left to evaporate. For 3, the PEEK edge around the electrode surface was wiped with a tissue, and the surface was dried with a gentle flow of N_2(g)_. Upon evaporation of water the electrode was placed directly into the cell containing Ar-sparged buffer. Electrochemical measurements containing molecular 1 and 2 was performed for solutions containing 0.47–1 mM cobaloxime in pH 7. The voltammogram of 3 was collected using batch C indicated in Table S1.†

## Data availability

The data supporting this article have been deposited in Open Science Framework repository, https://osf.io/mdpjq/?view_only=8f49e73a4b2d463092eb84c0f0bbcff4.

## Author contributions

Investigation of catalytic performance was carried out by S. B., C. B., and S. D. G. Electrochemical investigation was performed by S. B. Spectroscopic investigation was carried out by S. B. (UV-VIS), I. K. (MALDI), V. S. (ICP), A. M. (EPR), S. D. G. (CD). P. E. A. provided MALDI as a project resource. C. T. and M. L. prepared α_3_W for the CD investigation. Formal analysis of the data was performed by S. B., I. K., V. S., A. M., S. D. G., C. T. and M. L. S. B., C. T., A. M., and S. D. G. wrote the manuscript. S. D. G. and A. M. supervised the research. S. D. G. conceptualized, funded, and administered the project.

## Conflicts of interest

There are no conflicts of interest to declare.

## Supplementary Material

DT-053-D4DT00936C-s001
