# Supplementary material for: Hydrogen production by a fully de novo enzyme
Source: Dalton Trans. 2024 Jun 13;53(31):12905–16. doi: 10.1039/d4dt00936c (PMC11301571; doi:10.1039/d4dt00936c)
Supplement: DT-053-D4DT00936C-s001 [file DT-053-D4DT00936C-s001.pdf]

Electronic Supplementary Material (ESI) for Dalton Transactions.
This journal is © The Royal Society of Chemistry 2024

Supplementary Information

for:

Hydrogen production by a fully de novo enzyme

Sigrid Berglund[a], Clara Bassy[a], Ibrahim Kaya[b], Per E. Andrén[b], Vitalii Shtender[c] Mauricio Lasagna[e], Cecilia Tommos[e], Ann Magnuson[d] and Starla D. Glover*[a]

[a] Physical Chemistry, Department of Chemistry, Ångström Laboratory, Uppsala University, Box 523, SE-75120, Uppsala, Sweden, E-mail: Starla.glover@kemi.uu.se
[b] Department of Pharmaceutical Biosciences, Spatial Mass Spectrometry, Science for Life Laboratory, Uppsala University, Box 591, SE-75124 Uppsala, Sweden
[c] Division of Applied Materials Science, Department of Materials Science and Engineering, Uppsala University, 75103 Uppsala, Sweden
[d] Molecular Biomimetics, Department of Chemistry, Ångström Laboratory, Uppsala University, Box 523, SE-75120, Uppsala, Sweden
[e] Department of Biochemistry and Biophysics, Texas A&M University, College Station, TX 77843, United States

Table of Contents

I.	α_3C protein sequence	S2
II.	Determination of protein concentration, Table S1	S2
III.	Determination of cobalt concentration by ICP–OES, Table S2	S2-3
IV.	Additional data, Figures S1-S4, Table S3	S4-5
V.	Calculation of free Eu(II) in solutions of Eu(EGTA)	S6
VI.	Determination of initial rates from hydrogen evolution measurements, Table S4, Figures S5-S6	S7-8
VII.	Reaction of cysteine functionalization with 3-Br-methyl-pyridine, Figure S7	S9
VIII.	Determination of cobalt concentration by PAR assay, Figure S8	S9-10
IX.	[Co(dmg)$_2$Cl2] binding control study with α_3W	S10
Table S1	Summary of protein and cobalt concentrations	S2
Table S2	Cobalt concentrations by ICP-OES	S3
Table S3	Voltametric peak potentials	S5
Table S4	Time intervals for determination of initial rates	S7
Figure S1	S1 MALDI-TOF spectrographs of complex 3	S4
Figure S2	UV-visible absorption spectra of **2**, **3**, α_3C and 3-MePy-α_3C	S4
Figure S3	Chemical denaturation of α_3W, α_3C and **3**.	S5
Figure S4	Normalised cyclic voltammograms of complex **2** and **3**	S5
Figure S5	Time interval determination for photoactivated HER-experiments	S7
Figure S6.	Hydrogen evolution detected by Clarke-type electrode	S8
Figure S7	Cysteine pyridine labeling reaction	S9
Figure S8	PAR assay for Co^{2+} concentration	S10

I. α_3C protein sequence

The cysteine that is functionalized with methylpyridine is shown underscored in bold text.

```
GSRVKALEEK  VKALEEKVKA  LGGGGRIEEL  KKKCEELKKK IEELGGGGEV KKVEEEVKKL  EEEIKKL
        10          20          30          40         50         60
```

II. Determination of protein concentration

The protein concentration was determined either by the Bradford assay, using the Bio-Rad Protein Assay kit II (Bio-Rad Laboratories inc.), or with the Pierce™ BCA Protein Assay Kit (Thermo Scientific). The Bio-Rad Protein Assay was not compatible with cobaloxime functionalized protein, **3**, so the BCA assay was used to determine the concentrations of protein in the product. As a standard protein solution, we used purified α_3W instead of the provided bovine serum albumin. The α_3W protein concentration was determined from the optical density at 280 nm and molar absorption coefficient of 5500 M^{-1} cm^{-1}. The BCA enhanced test-tube procedure was performed as described, except that all volumes were halved in comparison to the provided instructions.

By comparing the protein quantification and the cobalt quantification (*vide infra*), it was found that the cobalt:protein ratio is ranged from 1:1.20-1:2.68. More specifically for the three batches of protein indicated here, the average cobalt:protein ratio was 1:1.79 (Table S2). The colorimetric Pierce™ BCA Protein Assay is less sensitive than ICP-OES which is used to determine the cobalt concentration.

Table S1. Comparison of cobalt and protein concentration for three independently prepared batches of **3**.

Sample	[Co] (M)	[Co] RSD (%)	[3-MePy-α_3C] (M)	Standard curve R^2	Cobalt:protein ratio
A	$3.53*10^{-5}$	0.4	$5.31*10^{-5}$	0.9999	1:1.50
B	$1.45*10^{-4}$	0.9	$3.88*10^{-4}$	0.9999	1:2.68
C	$5.76*10^{-4}$	0.4	$6.89*10^{-4}$	0.9999	1:1.20

III. Determination of cobalt concentration by ICP–OES.

The cobalt content in samples containing **2** and **3** was quantified using inductively coupled plasma – optical emission spectrometry (ICP–OES). This method was used for all samples of **2** and **3** subject to chemical reduction by $[Eu(EGTA)]^{2-}$; the cobalt concentration in samples used for photocatalysis and electrochemistry were subject to the assay described in section IV below. After the termination of each hydrogen evolution experiment the solution was sampled by ICP-OES. The concentration and relative standard deviation (RSD) for each sample is given in Table S1 with an identification number. These identification numbers are correlated to the numerical labels shown in Figure S6.

The ICP-OES experiment was performed with the Avio 200 ICP-optical emission spectrometer, (Perkin Elmer, Inc.). The presence of Co was confirmed by the emission at 228.616 nm where the peak was defined by three points. The samples for ICP-OES analysis were prepared by mixing equal volumes of the sample solution and 4 wt% concentrated nitric acid in milliQ water. The diluted samples were filtered with a 0.2 μm syringe filter. The sample cobalt concentrations were quantified against a calibration curve prepared from a commercial standard solution CPAchem of cobalt (100 mg/L Co in 2% HNO_3 matrix). Standards of lower concentration were prepared by appropriate dilution. The resulted calibration line described with 0.999918 correlation coefficient. All measured concentrations used to report quantities have RSD <3%.

Table S2. *In situ* cobalt concentration in each sample used for hydrogen evolution experiments, quantified by ICP-OES. Figure S6 shows sample number for each individual trace. Complex **2** = [Co(dmg)$_2$(py)Cl] and complex **3** = [Co(dmg)$_2$(3-MePy-α$_3$C)Cl]. Entries for [Co] with multiple values correspond to repeat measurements with average given in parentheses.

Sample number	Complex	pH	[Co] (μM)	RSD (%)
1	**2**	7	3.033	0.5
2	**2**	7	2.902	0.2
3	**2**	7	2.979	1.2
4	**3**	7	2.630/2.546 (2.587)	0.7/2.6
5	**3**	7	2.598	0.9
6	**3**	7	3.383	0.3
7	**3**	7	2.450	1.9
8	**2**	8	2.583	1.9
9	**2**	8	2.902	1.1
10	**2**	8	2.924	0.7
11	**2**	8	3.087	1.1
12	**3**	8	3.354	1.0
13	**3**	8	3.404	0.6
14	**3**	8	3.507	0.1
15	**3**	8	3.286	0.3
16	**3**	7	3.239/2.887 (3.062)	0.7/2.4
17	**3**	7	5.968	0.1
18	**3**	7	3.239	0.7

IV. Additional data

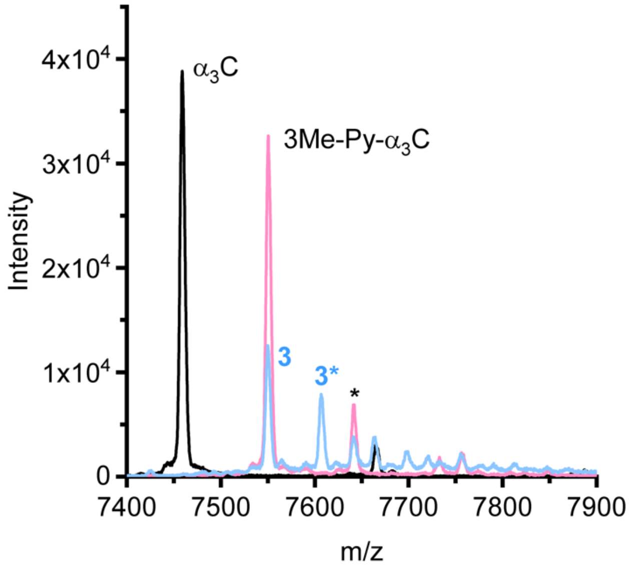

Figure S1 MALDI-TOF spectrographs of different protein samples: α_3C (black) (m/z = 7458.987), 3-MePy-α_3C (pink) (m/z = 7550.461) and **3** (blue) (m/z 3: 7550.144, m/z 3* = 7641.38). * = adducts m/z = 7606.725

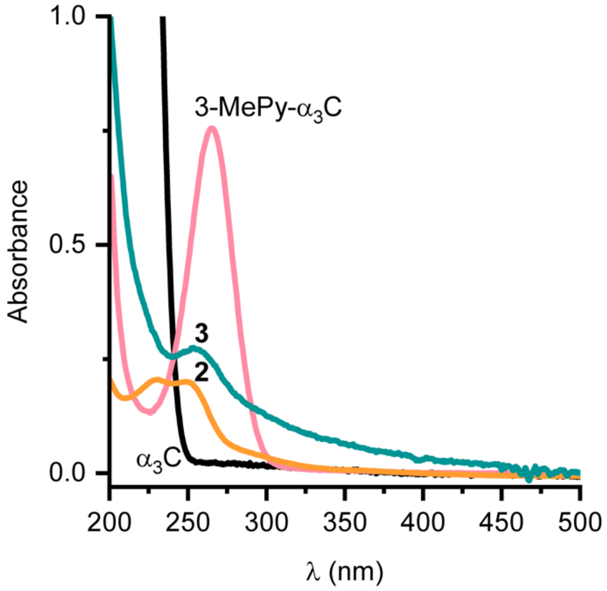

Figure S2. UV-visible absorption of 220 μM α_3C in 50 mM KP_i pH 7, 4 mm pathlength (black), 3 μM 3-MePy-α_3C in 50 mM KP_i pH 6, 10 mm pathlength (pink,), 40 μM **2** in water, 2 mm pathlength (orange), and 50.5 μM **3** in 12.5 mM KP_i pH 7, 1 mm pathlength (teal).

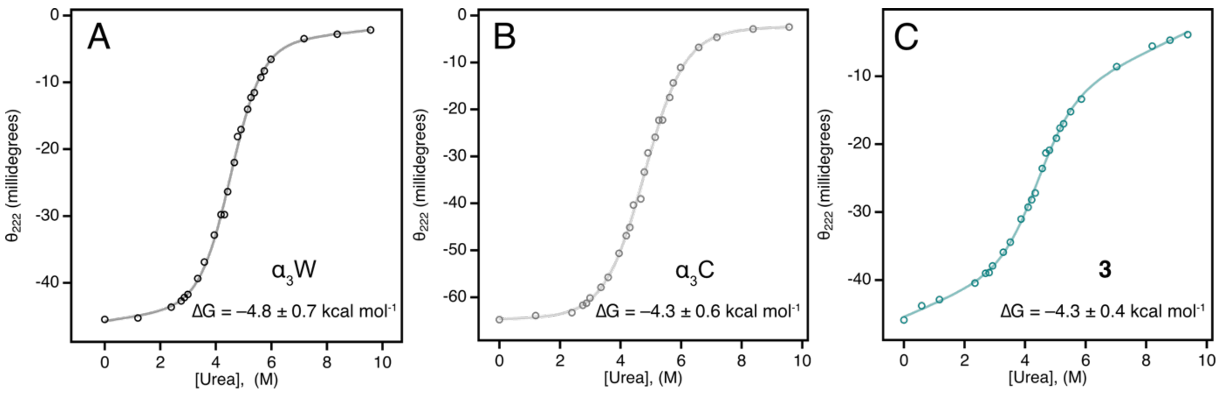

Figure S3. Chemical denaturation of α_3W, α_3C and **3** in 50 mM KP_i pH7**.** Folding/unfolding transition induced by addition of urea to: **A)** a sample of 19.1 µM α_3W, B) a sample of 18.8 µM α_3C, and C) a sample of 30.6 µM **3** (from Sample C, Table S1). Circles are averaged data obtained from three replicate spectra. Grey lines are non-linear curve fits used to determine the stability of the protein in the absence of denaturant.[3]

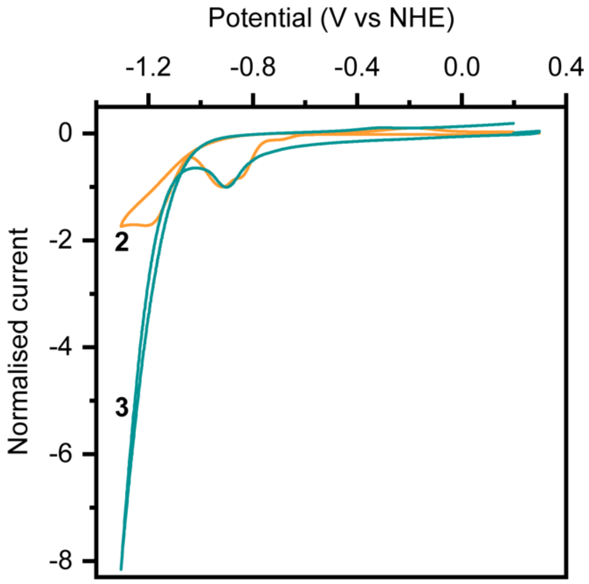

Figure S4: Normalised cyclic voltammograms of **2** (orange) and **3** (teal) (from figure 2D). The voltammograms were normalised to the peak currents at -0.920 V vs NHE (**2**) and -0.900 V vs NHE (**3**).

Table S3. *Voltametric peak potentials for* ***2*** *and* ***3****, from the cyclic voltammograms in figure 2D.*

Peak	**2**	**3**
Major anodic peak (V vs NHE)	-0.91811	-0.89736
Catalytic onset potential (V vs NHE)	-1.0469	-1.05941
Catalytic plateau (V vs NHE)	-1.20284	NA

V. Calculation of free Eu(II) in solutions of Eu(EGTA)

Ethylene glycol tetraacetic acid, EGTA, is a tetradentate ligand with four groups subject to protonation. The pK_a values associated with these groups are pK_1-pK_4 = 2.0, 2.68, 8.85, 9.43.[4] The concentration of $EGTA^{4-}$, the form which binds Eu(II), depends on the buffer pH. The concentration of $EGTA^{4-}$, can be determined using equation 14 from reference 3:

$$\alpha_H=\frac{(EGTA)'}{(EGTA)}=1+[H]\cdot 10^{pK_4}+[H]^2\cdot 10^{pK_4+pK_3}+[H]^3\cdot 10^{pK_4+pK_3+pK_2}+[H]^4\cdot 10^{pK_4+pK_3+pK_2+pK_1}$$

where α_H is the partition coefficient at a given hydrogen ion concentration, EGTA´ is the total amount of EGTA not bound to metal and (EGTA) is the total concentration of dissociated anion. The log of the stability constant, $logK_{EuEGTA}^{Eu(II)}$, was reported from electrochemistry to be 9.38.[5] Equation 15 from reference 3 is used to determines the apparent stability constant as a function of pH:

$$(K_{EuEGTA}^{Eu(II)})_{pH}=\frac{K_{EuEGTA}^{Eu(II)}}{\alpha_H}=\frac{(EuEGTA)}{(Eu)(EGTA)'}$$

where (EuEGTA) is the concentration of $[Eu(EGTA)]^{2-}$ in solution. Then using equation 6 from reference 3 the fraction of free metal in the solution can be calculated.

$$(Eu)=-\frac{1}{2}\cdot\left((EGTA)_t-(Eu)_t+\frac{1}{K_{EuEGTA}^{Eu}}\right)+\left(\frac{1}{4}\cdot\left((EGTA)_t+(Eu)_t+\frac{1}{K_{EuEGTA}^{Eu}}\right)^2+(Eu)_t\cdot\frac{1}{K_{EuEGTA}^{Eu}}\right)^{\frac{1}{2}}$$

where $(ETGA)_t$ and $(Eu)_t$ are the total concentration added to the solution. Finally one can calculate the concentration of $[Eu(EGTA)]^{2-}$ from $(Eu)_t$=(Eu)+[Eu(EGTA)].

VI. Determination of initial rates from hydrogen evolution measurements

Table S4. *Time intervals for determination of initial rates, under given experimental condition.*

Experiment	Time interval (min)	Notes
2 pH 6	40-80	Photoactivated HER, fig 3
3 pH 6	40-80	Photoactivated HER, fig 3
2 pH 7	1.37-2.00	Eu(EGTA)-activated HER, fig 4
3 pH 7	2.50-4.00	Eu(EGTA)-activated HER, fig 4
2 pH 8	0.81-1.80	Eu(EGTA)-activated HER, fig 4
3 pH 8	1.01-3.00	Eu(EGTA)-activated HER, fig 4

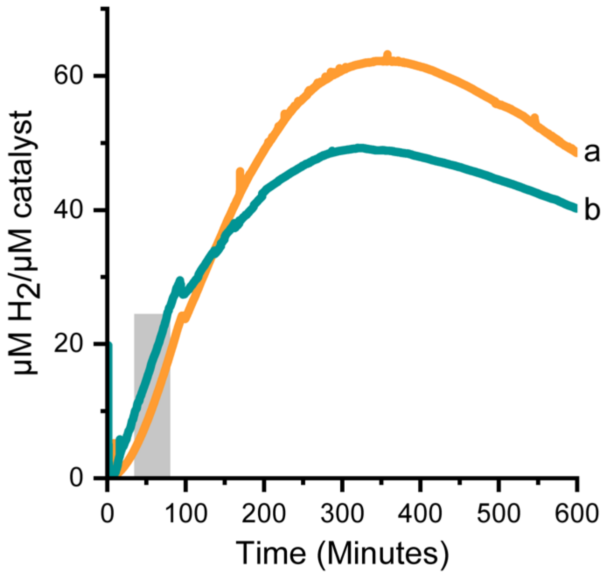

Figure S5. *Photochemical hydrogen evolution detected by Clark-type hydrogen sensor.* Figure 2B showing H_2 produced by **2 (a, orange)** and **3 (b, teal)** at pH 6.2 under photocatalytic conditions. Detection by H_2 sensing electrode. The shaded area shows which data were used for linear fits to determined rates of hydrogen production reported in Table 1.

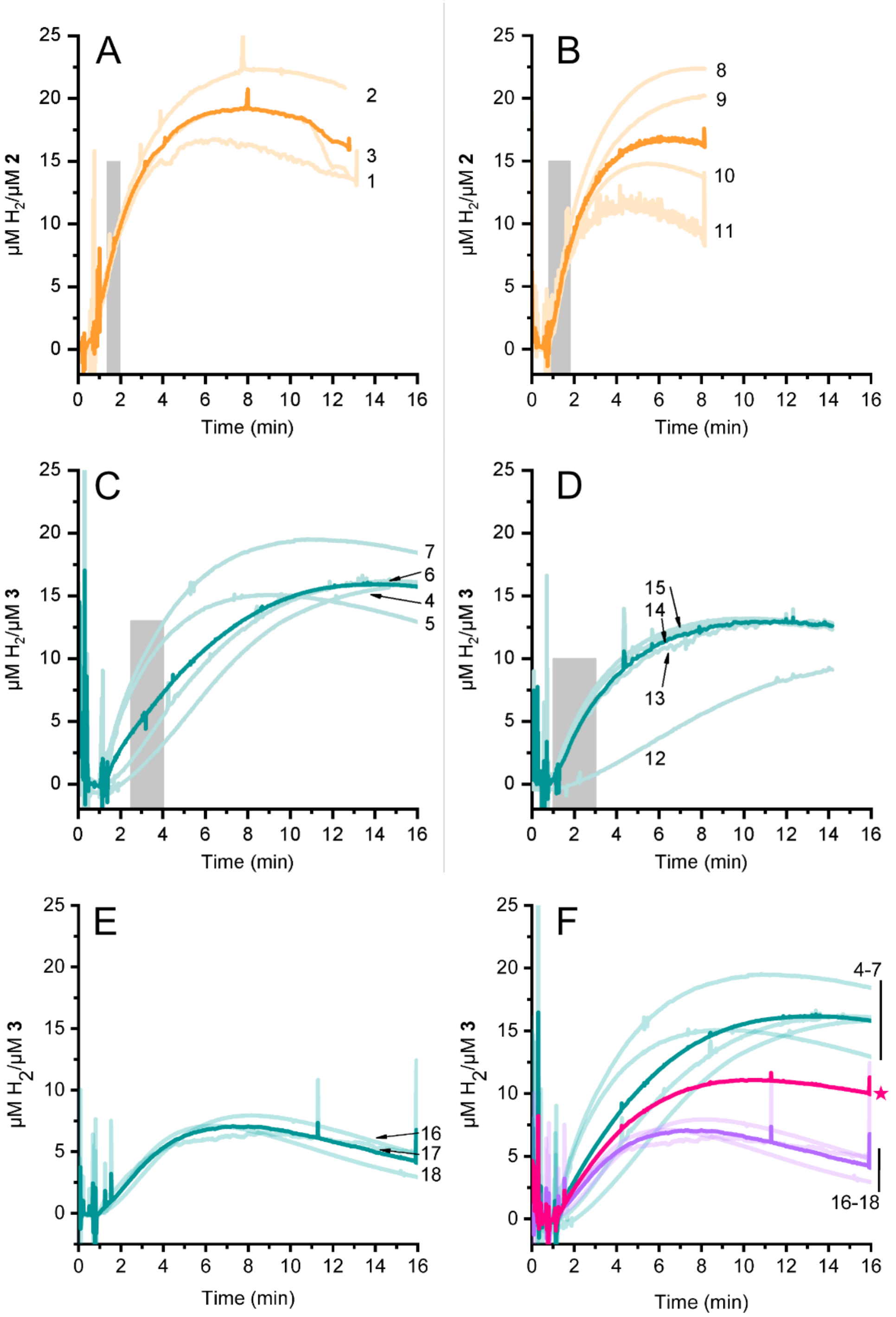

Figure S6. *Chemical hydrogen evolution detected by Clark-type hydrogen sensor.* Hydrogen evolved by **2** and **3** in the presence of chemical reducing agent $[Eu(EGTA)]^{2-}$. Light traces are individual measurements; dark traces are the average of repeated measurements. Traces 1-15 were recorded on the same day and therefore more directly comparable. Traces 16-18 were recorded on a different day. Numbers correspond to the sample number of Table S1. The shaded grey areas show which data were used for linear fits to determined rates of hydrogen production reported in Table 1. **A.** Complex **2**, pH 7. **B.** Complex **2**, pH 8. **C.** Complex **3**, pH 7. **D.** Complex **3**, pH 8. **E.** Complex **3**, pH 7 on different day. **F.** All hydrogen evolution traces (4-7 – teal, traces 4-7) and (16-18 – lilac, traces 16-18) for **3** at pH 7. The trace shown in pink (star) is the average of all purple and teal traces in the plot.

VII. Reaction of cysteine functionalization with 3-bromomethyl-pyridine

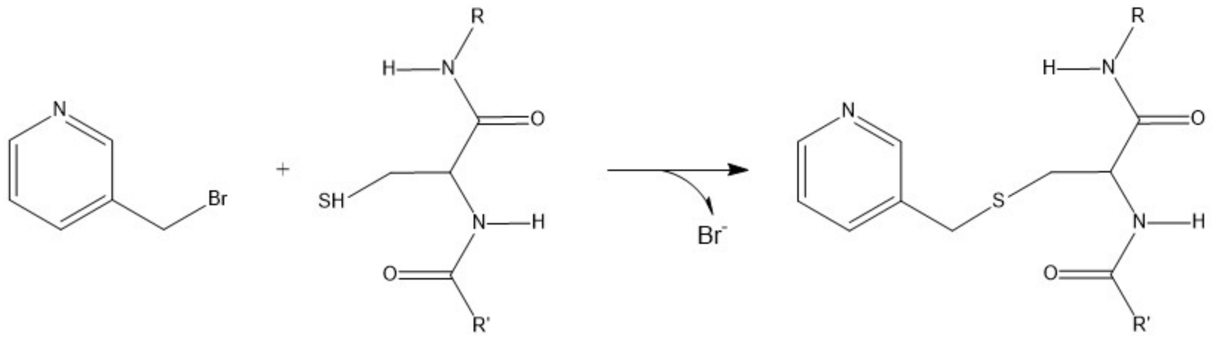

Figure S7. Reaction scheme showing covalent attachment of 3-methylpyridine to cysteine. Reaction was carried out in 2M guanidinium at pH 8.5 and resulted in a high yield of functionalized 3-MePy-α_3C as judged from MALDI (Figure 2A).

VIII. Determination of cobalt concentration by PAR assay

PAR, 4-(2-pyridylazo)resorcinol, is a ligand that forms colourful coordination complexes when bound to transition metal ions, such as Co^{2+}.[1,2] Summarized below is the colorimetric assay of PAR with varying concentrations of $CoCl_2$ or [Co(dmg$_2$)Cl$_2$]. A stock solution of 5 mM PAR was prepared in ethanol. The PAR stock was diluted to a concentration of 200 µM in 4 M aqueous Gdn:HCl. Calibration curves were determined by following the changes in optical spectra as known concentrations of $CoCl_2$ (Figures S8A, S8B) or [Co(dmg$_2$)Cl$_2$] (Figures S8C, S8D) were titrated into a solution of PAR. The standard curves were determined from the change in absorbance at 510 nm for $CoCl_2$ and [Co(dmg$_2$)Cl$_2$] in the concentration ranges of 0-20 µM, (Figures S8E). Standard curves for $CoCl_2$ versus [Co(dmg$_2$)Cl$_2$] differed by a factor of 2.03 at 412 nm and 2.43 at 510 nm. Samples quantified by $CoCl_2$ standard curve were checked against ICP-OES and differed by a factor of 3.5. The concentration of cobalt in samples containing **2** or **3** were determined by titration into 200 µM PAR in 4 M guanidinium, compared against the $CoCl_2$ calibration curve and multiplied by a factor of 3.5. This assay technique was used for all samples from photocatalysis.

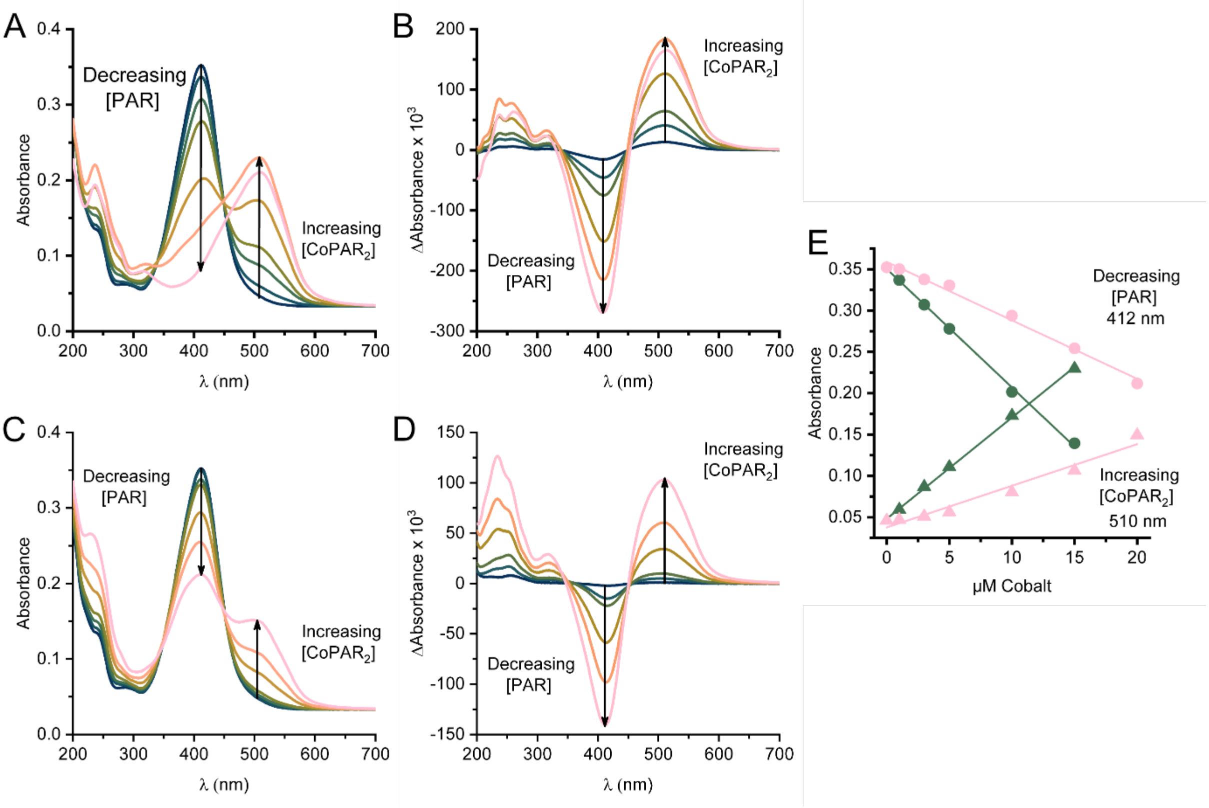

Figure S8. PAR assay for cobalt quantification. **A.** Spectra of PAR with increasing concentrations (0-20 μM) of $CoCl_2$ **B.** Difference spectra of $[Co(PAR)_n]^{2+}$ (n =1, 2) calculated by subtracting the spectrum in of PAR with no added cobalt from all other spectra. **C.** Spectra of PAR with increasing concentrations (0-20 μM) of $[Co(dmg)_2Cl_2]$. **D.** Difference spectra of $[Co(PAR)_n]^{2+}$ calculated by subtracting the spectrum of PAR with no added cobalt from all other spectra. **E.** Absorbance at 412 (circles) and 510 (triangles) nm as a function of $CoCl_2$ (green) and $[Co(dmg)_2Cl_2]$ (pink) concentration.

IX. Binding control - $[Co(dmg)_2Cl_2]$ and α_3W

We performed a binding control experiment using α_3W and $[Co(dmg)_2Cl_2]$, **1**, to test for potential cobaloxime binding to the protein scaffold that may occur at sites other than site 32. α_3W was treated with **1** under conditions identical to those used to bind **1** to 3-MePy-α_3C. Briefly, α_3W was dissolved in pH 8.5 buffer containing 2 M guanidinium. A 10-fold molar excess of $[Co(dmg)_2Cl_2]$ and 45-fold excess of reducing agent (TEA) were added to the protein solution and incubated under argon atmosphere for 4 hours at RT. The cobalt concentration in these samples was 0.87 mg/L. The reaction products were subsequently dialyzed against water in 3.5 kDA dialysis tubing. ICP analysis, with resolution of 0.0001 mg/L, was carried out on dialyzed protein samples. No cobalt was detected. From this experimental control we can conclude that under our experimental conditions, $[Co(dmg)_2Cl_2]$ does not bind to the non-coordinating amino acids in the α_3 scaffold.

References

1. C. E. Säbel, J. L. Shepherd and S. Siemann, *Anal. Biochem.*, 2009, ***391***, 74-76.
2. S. D. Glover and C. Tommos, in *Methods in Enzymology*, ed. A. J. Wand, Academic Press, **2019**, vol. 614, pp. 87-106.
3. M. M. Santoro and D. W. Bolen, *Biochemistry*, 1988, ***27***, 8063-8068.

4. J. Rafflaub, *Applications of Metal Buffers and Metal Idicators in Biochemistry*, Interscience Publishers, Inc., **1956**.
5. M. Botta, M. Ravera, A. Barge, M. Bottaro and D. Osella, *Dalton Trans.*, 2003, 1628-1633.
